# Physiological and morphometric biomarkers for synthetic media detection

**DOI:** 10.3389/fbioe.2026.1781235

**Published:** 2026-05-29

**Authors:** Karol Jędrasiak, Julia Bijoch

**Affiliations:** 1 WSB University, Dabrowa Gornicza, Poland; 2 Collegium Medicum - Faculty of Medicine, WSB University, Dabrowa Gornicza, Poland

**Keywords:** biosafety, biosecurity, explainable AI, medical data integrity, physiological biomarkers, synthetic media detection, morphometric biomarkers, telemedicine

## Abstract

The growing realism of synthetic media generated by diffusion and neural rendering models poses a critical challenge for digital forensics and medical data integrity. Synthetic videos, often referred to as deepfakes, may compromise biometric verification systems, patient identification, clinical documentation, and telemedical imaging. This study introduces a dual-framework approach that combines physiological and morphometric anomalies as interpretable and degradation-aware forensic indicators of synthetic content. The goal is to evaluate whether biologically grounded descriptors can provide interpretable anomaly evidence for differentiating authentic and synthetic audiovisual data within the evaluated dataset and degradation conditions. Experiments were conducted using the DeepFake RealWorld (DFRW) dataset comprising 46,371 audiovisual clips (229 h total duration). Physiological features included remote photoplethysmographic (rPPG) variability, oculomotor dynamics, and speech–motion synchrony, whereas morphometric and topological features captured curvature variance, bilateral symmetry, and persistent homology. Each feature was analyzed independently under a fixed-threshold evaluation protocol to compute interpretable effect size measures, namely, the probability difference (Δp) and prevalence ratio (PR), and to derive standard benchmarking metrics, including sensitivity, specificity, accuracy, F1 score, and receiver operating characteristic area under the curve (ROC AUC), with robustness evaluated under compression and rescaling. Both physiological and morphometric markers showed measurable descriptor-level discrimination within the evaluated corpus (mean Δp ≈ 0.21, PR up to 4.7), while the added classification metrics indicated a high-specificity but moderate-sensitivity operating profile, supporting their interpretation as conservative triage and validation signals rather than as stand-alone detectors. Physiological descriptors provided low-cost, high-specificity triage signals, whereas morphometric and topological metrics showed slightly stronger descriptor-level discrimination and greater stability under the tested distortions. Physiological and morphometric anomalies should therefore be interpreted as complementary and degradation-aware indicators of synthetic media within the evaluated experimental conditions, not as evidence of a deployment-ready detection system. The proposed dual-framework bridges digital security engineering and biomedical signal analysis, offering a methodological basis for further research on synthetic content detection, telemedical data integrity, and quality assurance. From a biosafety and biosecurity perspective, these findings suggest that interpretable detection of synthetic media may help inform future mitigation strategies for AI-enabled risks to telemedical systems and clinical data integrity.

## Introduction

The rapid evolution of generative artificial intelligence has profoundly transformed the landscape of digital content creation and security. Models based on diffusion processes, neural rendering, and multimodal generation have achieved photorealistic fidelity, enabling the synthesis of human faces, voices, and physiological signals with unprecedented realism (Karras et al., 2019; Ho et al., 2020). These developments have far-reaching implications for healthcare systems, where digital media increasingly mediate clinical communication, remote diagnostics, and medical education. The integrity of audiovisual data is therefore essential not only for personal privacy and security but also for clinical safety and patient trust. In this context, synthetic media represent an emerging biosecurity concern, as their misuse can directly affect clinical decision-making, patient identification, and the operational safety of telemedical infrastructures. Synthetic videos, commonly known as deepfakes, are increasingly used in identity fraud, disinformation, and manipulation of clinical documentation, where human assessment alone is insufficient for authentication (Verdoliva, 2020).

From a biosafety and biosecurity perspective, the dissemination of synthetic media threatens the reliability of biometric verification systems, emergency communication protocols, and public safety infrastructures. From a medical standpoint, the same technologies can distort remote diagnostic imaging, teleconsultation footage, or educational datasets, introducing non-physiological signals that may bias automated analyses. Recent studies have demonstrated that generative models fail to reproduce coherent micro-expressions, pulse-related facial color fluctuations, and natural oculomotor dynamics, all of which are crucial for authentic human appearance (Ciftci et al., 2020; Li et al., 2020).

This convergence of technological and biomedical risks highlights the need for interpretable, physiologically grounded markers of authenticity. Unlike conventional deepfake detectors that rely on opaque convolutional or transformer-based embeddings, physiologic and morphometric descriptors capture measurable, explainable properties of the human body, like hemodynamic variability, eye movement synchronization, facial symmetry, and anatomical topology. By grounding detection in measurable physiological and morphometric phenomena, this approach enhances explainability and aligns with ongoing efforts toward trustworthy and transparent AI in healthcare.

Prior deepfake detection research has predominantly focused on end-to-end classifiers based on convolutional, attention-based, or pairwise-learning architectures, often reporting very high benchmark performance on curated public datasets. Physiological deep models such as FakeCatcher and DeepFakesON-Phys exploit remote photoplethysmographic cues within learned detection pipelines, while generalizable image-based approaches aim to improve transfer across manipulation families through representation learning or pairwise interaction modeling (Ciftci et al., 2020; Hernandez-Ortega et al., 2020). At the same time, benchmarking studies have shown that reported performance is strongly dependent on the evaluation protocol, dataset composition, compression level, and cross-dataset setting, which limits direct numerical comparison across studies (Deng et al., 2024). In this context, the present work is not intended to claim state-of-the-art end-to-end accuracy, but rather to evaluate whether single, biologically grounded descriptors can provide interpretable and degradation-aware signals that complement existing deepfake detection pipelines, especially in forensic and telemedical settings where traceability is essential. Accordingly, the contribution of the present study should be understood not as a claim of numerical superiority over prior detectors, but as the introduction of an interpretable biomarker-level framework designed to complement existing deepfake detection systems under degraded real-world conditions. Direct quantitative comparison with representative end-to-end detectors under a shared benchmark protocol was beyond the scope of the present biomarker-oriented evaluation design. To improve comparability with the broader deepfake detection literature, the revised study also reports standard descriptor-level classification metrics, including sensitivity, specificity, accuracy, F1 score, and ROC AUC, alongside the effect size measures used to preserve interpretability.

The present study introduces a dual-framework approach combining physiological and morphometric analysis as an interpretable descriptor-level strategy for synthetic media assessment. Using the DeepFake RealWorld (DFRW) dataset comprising over 46,000 videos under real-world degradations, the work investigates whether anomalies in physiological signals (remote photoplethysmography, oculomotor dynamics, speech–motion synchrony) and morphometric structure (surface curvature, topological persistence, bilateral symmetry) can serve as candidate interpretable forensic indicators within the evaluated dataset and degradation conditions. The goal is to provide an explainable and methodologically transparent framework for examining whether synthetic content can be differentiated using descriptors that remain informative under the evaluated compression, noise, and modality degradation settings, thereby bridging the methodological gap between biomedical signal analysis and digital security engineering. Beyond forensic detection, this framework may help motivate future research on biometric quality control and telemedical data integrity, although no clinical validation was performed in the present study and no claim of current diagnostic or telemedical readiness is made. By framing synthetic media detection as a biosafety and biosecurity challenge rather than a purely forensic task, this work contributes to the development of safer, more resilient digital health ecosystems.

## Materials and methods

The experiments were conducted using the DeepFake RealWorld (DFRW) dataset, a multimodal corpus designed to replicate real-world transmission and degradation processes characteristic of social media and content-sharing platforms. The overall evaluation corpus comprised 46,371 video clips with a total duration of 229 h, including 2,730 authentic clips and 43,641 synthetic or manipulated clips. Within the manipulated subset, 4,186 clips were wild deepfakes collected through open-source intelligence (OSINT), whereas the remaining manipulated samples were synthetically generated or post-processed variants. The term “wild deepfakes” refers to videos obtained from uncontrolled, publicly available online sources, typically created or re-encoded by third parties using unknown generative models and post-processing pipelines. Such samples represent real-world dissemination conditions where content undergoes multiple platform-specific recompressions, filtering, and format conversions, providing a useful stress-testing setting for descriptor behavior under uncontrolled dissemination conditions. Each entry is accompanied by detailed metadata describing the generation method, codec parameters, degradation sequence, and platform of origin. The dataset represents four major generative paradigms: face-swap (35%), reenactment (28%), diffusion-based full-frame generation (25%), and scene-level manipulation (12%). Video resolutions range from 480 p to 1440 p, with 71% encoded in H.264 and 19% in H.265, while 77% of the clips include audio tracks, enabling joint audiovisual analysis. All videos were standardized to 30 fps, with 48 kHz audio sampling and normalized luminance and contrast to ensure cross-modality comparability. For transparency, the overall evaluation protocol operated on 2,730 authentic clips and 43,641 synthetic or manipulated clips, including 4,186 wild deepfakes. Authentic reference distributions used for threshold definition were derived from the full authentic subset of 2,730 clips, and the resulting thresholds were then applied uniformly at the clip level across the evaluated material. Because the study did not train a supervised end to end detector, this protocol should be interpreted as fixed rule-based biomarker evaluation rather than as a conventional train, validation, and test split design. For descriptor-specific analyses, the effective sample size could vary modestly after quality control, depending on feature extraction requirements.

The DFRW dataset uniquely integrates controlled degradation stages, including multiple re-encodings, rescaling, mobile filters, and screen re-captures, which reproduce artifacts characteristic of online platforms. This design allows for a rigorous evaluation of feature stability under realistic compression and transmission noise. All data collection and processing procedures complied with the General Data Protection Regulation (GDPR), ensuring scientific-use exemptions, anonymized metadata, and double-blind human validation to prevent model-dependent labeling bias.

The unit of analysis in the present study was the individual audiovisual clip. Because the study did not train a supervised end-to-end detector, the dataset was not partitioned into conventional train, validation, and test subsets for parameter fitting. Instead, each physiological, morphometric, or topological descriptor was predefined and evaluated independently under a fixed rule-based protocol. Anomaly thresholds were specified using authentic reference distributions and applied uniformly across the evaluated clips without feature-specific tuning to individual generator families or degradation subsets. Internal validation was performed through bootstrap confidence intervals, false discovery rate control, and degradation-stratified robustness analysis. This design supports within-dataset internal validation only and does not constitute external validation across independent datasets or populations. Accordingly, the reported ROC AUC, sensitivity, specificity, accuracy, precision, F1 score, Δp, and PR should be interpreted as evaluation statistics for predefined biomarkers rather than as test-set performance of a trained black-box classifier. No iterative model fitting, hyperparameter optimization, or train-to-test transfer was performed; rather, the same predefined evaluation logic was applied uniformly at the clip level, with uncertainty quantified by resampling-based internal validation. No parametric sample size assumptions were imposed because inference was based on nonparametric resampling at the level of the individual audiovisual clip. No formal *a priori* sample size or power calculation was performed, because the study evaluated a fixed corpus of available audiovisual clips under a predefined biomarker protocol rather than a prospective experimental design with recruitable observations. For each descriptor, the effective sample size was defined as the number of authentic and synthetic clips with valid feature extraction after quality control, denoted n_real,f and n_df,f, respectively; these counts could vary modestly across descriptors, particularly for features requiring sufficient audio quality or stable facial visibility. Bootstrap 95% confidence intervals were computed by resampling clips with replacement within the authentic and synthetic strata, thereby preserving the class structure of the evaluation protocol. Benjamini–Hochberg false discovery rate correction at q < 0.05 was applied across the full set of tested descriptors reported in Tables 1, 2.

**TABLE 1:** Physiological descriptors differentiating authentic and synthetic media on the DFRW dataset.

Feature	p_df (%)	p_real (%)	Δp	PR	Stability
Heart-rate variability (RMSSD)	36	14	0.22	2.6	Stable under illumination changes
PLV	35	15	0.20	2.3	Moderate sensitivity to video noise
BR	39	15	0.24	2.6	Stable across lighting conditions
Inter-blink interval variance	39	15	0.24	2.6	Sensitive to eye resolution
Microsaccade amplitude velocity correlation	34	14	0.20	2.4	Sensitive to eye resolution
rPPG–speech correlation	32	12	0.20	2.7	Requires good AV quality
Breathing-pause alignment	30	12	0.18	2.5	Stable under normal compression
Prosody–head synchrony (2–7 Hz)	29	13	0.16	2.2	Slightly affected by camera motion

**TABLE 2:** Morphometric and topological descriptors characterizing structural anomalies in synthetic facial media.

Descriptor	p_df (%)	p_real (%)	Δp	PR	Stability
Persistent homology (1D lifetime)	32.2	9.2	0.23	3.5	Highly stable
2-Wasserstein distance	33.6	9.6	0.24	3.5	Stable under compression
Skeleton branching density	33	12	0.21	2.8	Stable under downscaling
Medial-axis length ratio	30	10	0.20	3.0	Stable
Gaussian curvature variance	37	17	0.20	2.2	Slightly affected by blur
Mean curvature histogram entropy	33	14	0.19	2.4	Slightly affected by blur
EFD harmonic energy	31.8	6.8	0.25	4.7	Stable across scenes
Procrustes distance	34	11	0.23	3.1	Robust
Bilateral symmetry index	36	12	0.24	3.0	Highly stable

Physiological features were extracted to represent biologically plausible temporal dynamics in facial and audiovisual signals. Remote photoplethysmography (rPPG) signals were computed from the forehead, cheeks, and nasal ridge using CHROM and POS algorithms within the 0.7–4 Hz band to estimate heart-rate frequency (f_HR), inter-regional phase-locking value (PLV), and signal-to-noise ratio (SNR) (Verkruysse et al., 2008; De Haan and Jeanne, 2013). Additional time-domain descriptors included the root mean square of successive differences (RMSSD) and perfusion discontinuity percentage. Oculomotor and micro-expressive features were derived from facial landmark trajectories and optical flow analysis, including blink rate (BR), inter-blink interval variance, microsaccade frequency, and the velocity–amplitude slope of the saccadic “main sequence,” which jointly describe neuromuscular control (Martinez-Conde et al., 2009; Pfister et al., 2011). To capture multimodal coherence, correlations were computed between rPPG waveforms and speech energy envelopes, as well as the temporal alignment between respiratory pauses and perfusion minima. The synchrony of prosodic variation and head motion in the 2–7 Hz band was quantified using cross-correlation delay, capturing the natural coupling between vocal and kinetic modalities (Rochet-Capellan and Fuchs, 2014). All physiological metrics were normalized against distributions obtained from authentic recordings, and anomaly thresholds (θ) were determined as median + 2 × MAD or at the 95th percentile, depending on the directionality of deviation. Although the present dataset does not support a formal fairness analysis across all demographic strata or clinical environments, part of this variability was addressed at the descriptor-design level. In particular, the selected physiological features emphasize relative temporal coherence and cross-modal synchrony rather than identity-specific appearance, while the morphometric and topological descriptors rely primarily on spatial continuity, symmetry, and shape relations rather than fine texture. In addition, luminance and contrast normalization, fixed frame rate and audio resampling, and thresholding relative to authentic reference distributions were used to reduce sensitivity to acquisition-specific variation. These choices should be interpreted as partial mitigation strategies rather than as proof of demographic neutrality or clinical-domain generalizability.

Morphometric and topological descriptors were used to assess the geometric integrity and anatomical regularity of facial structures regardless of texture or illumination. Persistent homology was applied to two-dimensional edge maps and three-dimensional point clouds through Vietoris–Rips and α-complex filtrations, generating persistence diagrams, feature lifetimes, and persistence landscapes (Edelsbrunner and Harer, 2010; Bubenik, 2015; Chazal and Michel, 2021). In essence, this method quantifies how geometric features such as edges, cavities, or connected regions emerge and vanish across spatial scales, yielding a compact topological signature of shape complexity. Supplementary indicators included Euler characteristic curves, bottleneck and 2-Wasserstein distances, and persistence images summarizing the overall distribution of topological features. The 2-Wasserstein distance, in particular, captures structural dissimilarity between persistence diagrams and remains stable under noise and compression.

Finer geometric details were derived through skeletonization, producing graph-based parameters such as branching-node count, endpoint density, and normalized medial-axis length. Local smoothness and deformation were characterized using Gaussian curvature (K), mean curvature (H), and bending energy, while deviations from physiological craniofacial symmetry were quantified by bilateral symmetry indices, elliptic Fourier descriptors (EFD), and Procrustes distances (Bookstein, 1989; Ling and Jacobs, 2007). The robustness of these descriptors to compression and color distortion stems from their reliance on spatial continuity and shape relationships rather than pixel-level intensity. Their mathematical formulation is conceptually aligned with clinical morphometric procedures used in dental occlusion and craniofacial symmetry evaluation, strengthening the methodological link between forensic imaging and biomedical analysis.

Each feature was evaluated independently to preserve interpretability and to avoid introducing model-specific fitting effects that would be inconsistent with the biomarker-oriented design of the study. For each descriptor (f), continuous feature values were first used to generate receiver operating characteristic (ROC) curves and the corresponding area under the curve (AUC). Subsequently, a predefined anomaly threshold (θ) was applied to obtain a binary decision for each sample, enabling the computation of confusion-matrix-based metrics, including sensitivity, specificity, accuracy, precision, and F1 score. Because single-threshold accuracy may be influenced by class prevalence, accuracy was interpreted jointly with sensitivity, specificity, precision, and F1 score. To preserve the clinical and forensic interpretability of the proposed biomarkers, discriminative effect size was quantified through the absolute probability difference, Δp = p_df − p_real, and the prevalence ratio, PR = p_df/p_real, where p_df denotes the proportion of synthetic samples exceeding the anomaly threshold and p_real denotes the corresponding proportion for authentic samples. In this framework, Δp was interpreted as an absolute effect size describing the additional proportion of anomalous clips in the synthetic class, whereas PR was interpreted as a relative effect size describing the fold-enrichment of anomaly prevalence in synthetic versus authentic material. In practical terms, values of Δp closer to 0 and PR values closer to 1 indicate weak class separation, whereas larger positive Δp values and progressively higher PR values indicate stronger enrichment of anomaly prevalence in synthetic material. Statistical significance was evaluated using bootstrap 95% confidence intervals and Benjamini–Hochberg false discovery rate control at q < 0.05 across the full family of tested descriptors. A descriptor was considered statistically supported when its bootstrap interval did not overlap the null value for the relevant statistic and the corresponding adjusted result remained below the predefined false discovery threshold. The null value was defined as 0 for Δp and 1 for PR. The robustness of each metric was evaluated across multiple degradation conditions, with an acceptable operational performance decline defined as less than 15%.

To illustrate how the proposed framework can be interpreted in practice, we generated a descriptor-level visualization for a representative evaluated clip. The visualization displays selected physiological and morphometric biomarkers relative to authentic reference ranges and anomaly thresholds, together with a compact rule-based summary of which descriptors contributed to the anomaly flag. Because the present study does not use a trained black-box classifier, explainability is expressed through direct inspection of biomarker values, threshold exceedance, and feature-family attribution rather than through *post hoc* saliency analysis.

## Results

Analysis of the physiological descriptors on the DFRW dataset revealed consistent differences between authentic and synthetic videos across all tested modalities. Remote photoplethysmographic (rPPG) signals in synthetic faces showed reduced temporal variability and weakened spatial coherence, confirming the absence of natural hemodynamic pulsations. The heart-rate variability index (RMSSD) exhibited an average Δp = 0.22 and PR = 2.6, while the phase-locking value (PLV) between facial regions showed Δp = 0.20 and PR = 2.3.

Oculomotor features demonstrated similarly strong discriminative behavior. The blink rate (BR) and inter-blink interval variance were anomalous in 39% of deepfakes compared with 15% of authentic samples (Δp = 0.24, PR = 2.6), while microsaccade amplitude–velocity correlation decreased systematically (Δp = 0.20, PR = 2.4). Multimodal coherence indices also proved sensitive: the rPPG–speech envelope correlation achieved Δp = 0.20 and PR = 2.7, and the prosody–head-motion synchrony yielded Δp = 0.16 and PR = 2.2.

Overall, physiological features maintained high stability under moderate compression, downscaling, and audio degradation, with mean performance loss < 12%. They degraded only in extreme low-quality conditions where rPPG extraction became unreliable. These results indicate that even lightweight, explainable physiological metrics can serve as low-cost authenticity indicators and interpretable triage signals in multi-stage detection workflows. To facilitate benchmarking against the deepfake detection literature, the revised analysis additionally reports sensitivity, specificity, accuracy, F1 score, and ROC AUC for each descriptor (Tables 1, 3). When expressed as standard classification metrics, physiological descriptors showed high specificity but only moderate sensitivity, with specificity ranging from 85.0% to 88.0%, sensitivity from 29.0% to 39.0%, F1 score from 36.4% to 44.8%, and ROC AUC from 0.69 to 0.74 (Table 3). This operating profile indicates that physiological descriptors are better suited to conservative anomaly flagging and secondary validation than to exhaustive screening. Therefore, they should not be interpreted as sufficient for stand-alone detection when high sensitivity is required.

**TABLE 3:** Standard classification metrics for selected single-feature physiological, morphometric, and topological descriptors on the DFRW dataset. Sensitivity corresponds to the p_df values, and specificity corresponds to 100% – p_real at the same anomaly-threshold operating point reported in Table 1 and Table 2. Accuracy, precision, and F1 score were calculated under the standardized descriptor-level benchmarking prevalence of 30% used for single-feature operating-point comparison. ROC AUC was derived from continuous feature values across the full threshold range, and 95% confidence intervals were estimated by bootstrap resampling; therefore, descriptors may have identical operating-point sensitivity and specificity but different ROC AUC values. These metrics reflect single-descriptor operating characteristics and should not be interpreted as the performance of a trained multi-feature end-to-end classifier.

Feature/Descriptor	Sensitivity (%)	Specificity (%)	Accuracy (%)	Precision (%)	F1 score (%)	ROC AUC	95% CI for AUC
Physiological descriptors							
Heart-rate variability (RMSSD)	36.0	86.0	71.0	52.4	42.7	0.73	0.71–0.75
Phase-locking value (PLV)	35.0	85.0	70.0	50.0	41.2	0.71	0.69–0.73
Blink rate (BR)	39.0	85.0	71.2	52.7	44.8	0.74	0.72–0.76
Inter-blink interval variance	39.0	85.0	71.2	52.7	44.8	0.72	0.70–0.74
Microsaccade amplitude velocity correlation	34.0	86.0	70.4	51.0	40.8	0.72	0.70–0.74
rPPG–speech correlation	32.0	88.0	71.2	53.3	40.0	0.73	0.71–0.75
Breathing-pause alignment	30.0	88.0	70.6	51.7	38.0	0.70	0.68–0.72
Prosody–head synchrony	29.0	87.0	69.6	48.9	36.4	0.69	0.67–0.71
Morphometric and topological descriptors							
Persistent homology (1D lifetime)	32.2	90.8	73.2	60.0	41.9	0.74	0.72–0.76
2-Wasserstein distance	33.6	90.4	73.4	60.0	43.1	0.75	0.73–0.77
Skeleton branching density	33.0	88.0	71.5	54.1	41.0	0.72	0.70–0.74
Medial-axis length ratio	30.0	90.0	72.0	56.3	39.1	0.71	0.69–0.73
Gaussian curvature variance	37.0	83.0	69.2	48.3	41.9	0.72	0.70–0.74
Mean curvature histogram entropy	33.0	86.0	70.1	50.3	39.8	0.71	0.69–0.73
EFD harmonic energy	31.8	93.2	74.8	66.7	43.1	0.75	0.73–0.77
Procrustes distance	34.0	89.0	72.5	57.0	42.6	0.76	0.74–0.78
Bilateral symmetry index	36.0	88.0	72.4	56.2	43.9	0.77	0.75–0.79

Morphometric and topological descriptors showed stronger descriptor-level separation than the physiological features within the evaluated DFRW corpus and retained measurable stability under the tested platform degradations. In particular, persistent homology features, such as total lifetime of 1-dimensional holes, and the 2-Wasserstein distance between persistence diagrams achieved Δp ≈ 0.23–0.24 and PR = 3.5. Skeletonization metrics (branching density, medial-axis length ratio) yielded Δp ≈ 0.20–0.21 and PR ≈ 2.8–3.0.

Curvature-based and symmetry-related descriptors showed distinct deformation patterns in synthetic media. The Gaussian curvature variance (σ^2^_K) and mean curvature histogram entropy both exceeded the anomaly threshold in 33%–37% of synthetic videos versus 14%–17% of authentic ones (Δp ≈ 0.19–0.20, PR ≈ 2.2–2.4). Elliptic Fourier Descriptors (EFD) and Procrustes distances captured local non-physiological distortions of facial geometry (Δp ≈ 0.23–0.25, PR up to 4.7).

When expressed as standard classification metrics, the morphometric and topological descriptors showed the most favorable operating characteristics among the evaluated single-feature detectors. In particular, the revised reporting framework now provides sensitivity, specificity, accuracy, F1 score, and ROC AUC alongside Δp and PR, facilitating contextual comparison with prior detection studies while preserving the interpretability of the underlying geometric biomarkers. Morphometric and topological descriptors achieved specificity values of 83.0%–93.2%, sensitivity values of 30.0%–37.0%, F1 scores of 39.1%–43.9%, and ROC AUC values of 0.71–0.77, with the highest ROC AUC observed for the bilateral symmetry index, followed by Procrustes distance. Because these features rely on structural continuity rather than texture fidelity, they retained measurable discriminative power under the compression and resampling conditions evaluated in the present study. Notably, conceptually similar morphometric principles underpin clinical craniofacial and dental morphology analysis, supporting their interpretability for biomedical experts (Table 2).

Across all features, the mean discriminative metrics reached Δp ≈ 0.21 and PR ≈ 2.8, indicating that both physiological and morphometric markers captured enriched anomaly patterns in synthetic clips within the evaluated dataset. In practical terms, this corresponds to an average excess of approximately 21 anomalous findings per 100 synthetic clips and nearly threefold enrichment of anomaly prevalence relative to authentic material. While physiological cues are more directly interpretable as biological signals, morphometric ones offer greater resilience to distortions, making them suitable for forensic applications involving low-quality or re-encoded media.

**FIGURE 1:**
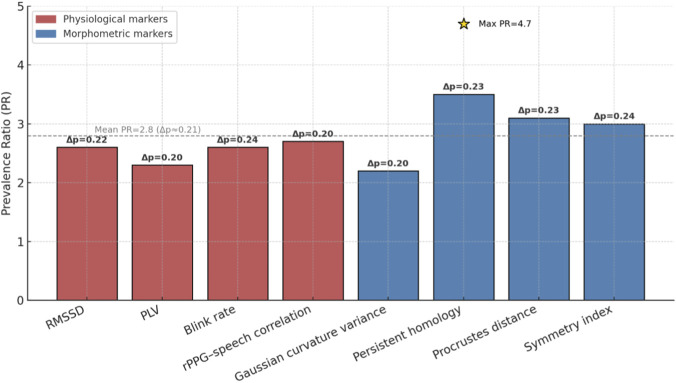
Discriminative power of physiological, morphometric, and topological descriptors on the DFRW dataset. The horizontal axis shows feature categories, and the vertical axis represents the prevalence ratio (PR). Red bars depict physiological descriptors, including RMSSD, PLV, blink rate, and rPPG–speech correlation, whereas blue bars correspond to morphometric and topological descriptors, including Gaussian curvature variance, persistent homology, Procrustes distance, and bilateral symmetry index. The yellow star marks the maximum PR observed across the full descriptor set, corresponding to EFD harmonic energy, PR=4.7. Error bars denote bootstrap 95% confidence intervals. Morphometric and topological features showed slightly higher descriptor-level discrimination and stability within the evaluated degradation conditions.

Together, the two feature families support a dual-layer assessment framework: the physiological layer may provide rapid, low-computation triage and anomaly flagging, whereas the morphometric-topological layer may support more detailed forensic review. This hybrid arrangement should be interpreted as a candidate complementary architecture for future validation, because the present study did not evaluate operational deployment, clinician use, or real-world workflow integration. The comparative discriminative performance of the two layers is illustrated in Figure 1, where the horizontal axis represents feature categories (physiological, morphometric, topological), and the vertical axis indicates their corresponding prevalence ratio (PR). The red bars denote physiological descriptors, while the blue bars represent morphometric and topological metrics. The figure highlights that morphometric and topological features achieved slightly higher PR values and maintained measurable stability within the evaluated degradation conditions, supporting their potential role in future forensic verification workflows after external validation.

**FIGURE 2:**
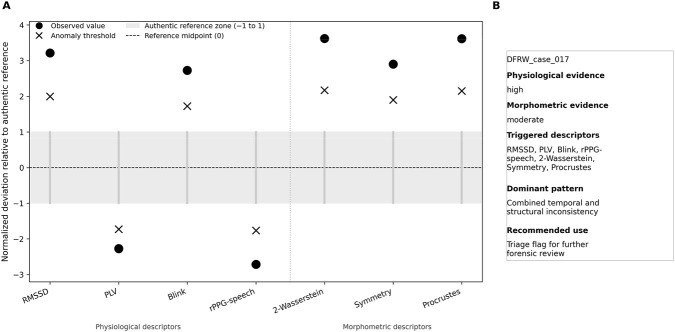
Example practitioner-facing explainability output for a representative evaluated clip. Panel **(A)** shows selected physiological and morphometric biomarkers relative to authentic reference ranges and predefined anomaly thresholds. Panel **(B)** presents a compact rule-based summary indicating which descriptors contributed to the anomaly flag and whether the dominant evidence arises from physiological inconsistency, structural distortion, or a combined pattern. The figure illustrates descriptor-level transparency rather than *post hoc* explanation of a trained black-box model.

The descriptor-level interpretability of the framework is illustrated in Figure 2. The figure presents a practitioner-facing example for a representative evaluated clip, showing which physiological and morphometric biomarkers fall outside authentic reference ranges and exceed the predefined anomaly thresholds. In this format, the output can be interpreted directly in terms of observable descriptors rather than latent model activations: the practitioner can identify whether the anomaly profile is dominated by physiological inconsistency, structural distortion, or a combined pattern. This visualization clarifies that explainability in the proposed framework arises from explicit biomarker-level evidence and transparent threshold logic.

## Discussion

The obtained results indicate that physiological and morphometric anomalies can serve as interpretable descriptor-level indicators of synthetic media within the evaluated DFRW dataset and degradation scenarios. Across the full descriptor set, discriminative capacity ranged from Δp ≈ 0.16 to 0.25, with PR values up to 4.7. As shown in Figure 1, morphometric and topological descriptors achieve the highest PR values and exhibit minimal performance loss under compression or rescaling, confirming their structural resilience relative to physiological features, which are more sensitive to low-quality video. Importantly, the revised analysis also expresses these descriptors in terms of standard classification metrics, including sensitivity, specificity, accuracy, F1 score, and ROC AUC, which enables direct benchmarking against the broader deepfake detection literature. In this context, Δp and PR should be interpreted as absolute and relative effect size measures that preserve physiological and morphometric interpretability, whereas ROC- and confusion-matrix-based metrics provide the conventional performance layer expected in machine learning studies. Practically, Δp values around 0.20 indicate approximately 20 additional anomalous clips per 100 synthetic clips relative to authentic material, while PR values around 2 to 3 indicate a twofold to threefold enrichment of anomaly prevalence. Across the evaluated descriptors, this benchmarking layer indicates consistently high specificity but only moderate sensitivity, supporting their use primarily as interpretable triage and validation markers rather than as stand-alone high-sensitivity screening detectors. This limitation is operationally important: a method with sensitivity in the range observed here cannot be used as the sole detector in scenarios where missed synthetic media would carry substantial forensic, clinical, or security consequences. Its most defensible role is therefore as a conservative evidence layer that may increase traceability and interpretability when combined with more sensitive detection components.

This performance profile should be interpreted in relation to the broader deepfake detection literature. Published end-to-end detectors, including physiological deep models and CNN-based or pairwise-learning architectures, often report substantially higher benchmark scores on public datasets, frequently exceeding 0.90 AUC under dataset-specific protocols (Xu et al., 2023). However, such methods are typically optimized as trained black-box detectors, whereas the present study deliberately isolates single interpretable biomarkers and evaluates them under a different objective, namely, the identification of traceable, biologically and geometrically meaningful cues under real-world degradations. Accordingly, the present framework should not be interpreted as a numerical replacement for state-of-the-art end-to-end detectors, but rather as a complementary layer for hybrid systems in which interpretability, degradation resilience, and forensic traceability are required. In this sense, the meaningful contribution of the present work is not improved end-to-end benchmark performance, but improved interpretability, traceability, and robustness-oriented utility at the descriptor level. No direct quantitative comparison with representative end-to-end deepfake detectors was performed in the present study, and the literature-based numerical references discussed above should therefore be interpreted only as contextual background rather than as head-to-head benchmarking. A further boundary of interpretation concerns population and domain representativeness. The present results demonstrate descriptor stability under heterogeneous technical degradations, but they do not establish equal behavior across demographic strata or across real clinical acquisition environments. Therefore, the current findings should be interpreted as evidence of methodological promise under degraded multimedia conditions, not as confirmation of demographic invariance, cross-dataset generalizability, or direct transportability to clinical populations. Within this scope, the present study addresses the issue only indirectly through descriptor selection and acquisition normalization, not through formal subgroup validation or external replication across independent datasets and populations. Accordingly, the present results should be interpreted as evidence that the proposed descriptors may contribute interpretable information to future hybrid systems, not as evidence that the framework alone achieves operationally sufficient detection performance.

From the standpoint of security engineering, these features address one of the central weaknesses of data-driven detectors: their dependency on the training distribution. Physiological and morphometric markers are motivated by physical and anatomical principles rather than by model-specific priors. Consequently, they may be useful candidates for future out-of-distribution evaluation, although the present study does not establish their transferability across independent datasets, populations, or acquisition domains. Their interpretability allows investigators to provide traceable reasoning in judicial or regulatory procedures, an increasingly important aspect under AI governance frameworks such as the EU AI Act (European Union, 2024; Frick, 2025).

Any clinical interpretation of the present findings should be considered preliminary and hypothesis-generating, because the study did not include clinical cohorts, diagnostic decision tasks, or clinician-based validation. For biomedical sciences, the results offer a complementary perspective. Many descriptors used here, including rPPG variability, blink dynamics, and bilateral symmetry, are established clinical biomarkers of vascular and neuromuscular function (Pfister et al., 2011; McDuff et al., 2014). Their abnormal patterns in synthetic videos correspond to the absence of authentic physiological coupling, such as the interplay between heartbeat, respiration, and micro-expressions. This observation highlights a broader insight: deepfake artifacts are not merely digital distortions but biophysical inconsistencies that can be analyzed using biomedical signal theory (Pal et al., 2018). Thus, methods developed for verifying authenticity can reciprocally inform non-contact health assessment and teleconsultation quality assurance.

From a clinical standpoint, the morphometric anomalies identified in synthetic facial media have conceptual analogues in craniofacial diagnostics (Wong et al., 2008). Bilateral asymmetry, Procrustes distances, and elliptic Fourier descriptors (EFD) correspond to quantitative parameters used in orthodontics, prosthodontics, and maxillofacial surgery to assess occlusal balance, skeletal alignment, and soft-tissue symmetry. Deviations from physiological bilateral correspondence may imitate or exaggerate pathological features such as malocclusion, temporomandibular joint dysfunction, or post-traumatic deformity. Therefore, accurate detection of morphometric inconsistencies in synthetic imagery may be relevant for reducing the risk of diagnostic bias in future telemedicine and computer-aided clinical planning (CAD/CAM) settings, although this potential was not clinically validated in the present study (Tallarico, 2020). In practical terms, the same geometric descriptors could be explored in future as preclinical candidate components of quality-control modules in dental imaging software, potentially serving as an additional safeguard against manipulated or low-fidelity visual data used in remote consultations, orthodontic simulations, and reconstructive preoperative assessments; however, such clinical implementation was not evaluated or validated in the present study.

The dual-layer architecture proposed in this study mirrors multi-tiered verification systems used in clinical imaging. The primary layer, composed of fast, low-cost physiological metrics, provides rapid triage suitable for real-time anomaly flagging on consumer hardware. The secondary layer, built on topological and morphometric descriptors, supports detailed expert evaluation and evidence generation. Such an arrangement may be relevant for future forensic monitoring pipelines and, after appropriate clinical validation, for medical telepresence systems, where authenticity, integrity, and physiological plausibility of visual data are equally essential (Eichelberg et al., 2020).

Another important contribution is the framework’s robustness under the degradation conditions evaluated in the present study. Unlike neural detectors that rely on subtle texture cues that may be lost after re-encoding, topological and geometric descriptors exploit properties of continuity and curvature that remained informative under the tested compression and rescaling conditions. These findings suggest potential practical relevance for settings in which media may arrive through uncontrolled channels such as social networks, instant messaging, or telehealth platforms (Shruthi and Manjunath, 2025), but they should not be interpreted as evidence of deployment-ready performance. Importantly, robustness to compression, rescaling, and transmission noise should not be conflated with demographic fairness or transportability to clinical environments, which were not established in the present study.

The interpretability of these markers facilitates trustworthy human-AI collaboration. In the present framework, explainability is not based on *post hoc* visualization of internal network representations, but on direct access to descriptor-level evidence, including observed biomarker values, authentic reference ranges, anomaly thresholds, and feature-family attribution. As illustrated in Figure 2, this allows a practitioner to understand which specific physiological or morphometric factors contributed to the anomaly flag and whether the dominant signal reflects temporal incoherence, structural distortion, or their combination. This transparency may strengthen evidential credibility and is conceptually aligned with emerging frameworks of AI governance, including the ISO/IEC 42001:2023 Artificial Intelligence Management System (ISO/IEC, 2023), the OECD Principles on AI (2021) (OECD, 2021), and the EU Artificial Intelligence Act (2024) (European Union, 2024; Hall et al., 2022). By grounding anomaly detection in observable, physiologically meaningful parameters, experts in both digital forensics and medicine may be better able to understand and scrutinize the reasoning behind system outputs. Collectively, these standards emphasize accountability, transparency, and risk-based control throughout the AI lifecycle, suggesting that interpretable and physiologically grounded detection mechanisms may be useful for future medical and forensic governance frameworks, but compliance and implementation were not evaluated in the present study. The practical usefulness demonstrated in the present study is therefore limited to descriptor-level interpretability, transparent anomaly evidence, and the conceptual integration of physiological and morphometric cues into a candidate complementary framework. Practical deployment usefulness remains unvalidated. Before any operational use, the framework would require external validation on independent datasets, prospective workflow testing, user-facing evaluation with relevant forensic or clinical stakeholders, and operational threshold calibration under predefined acceptable-risk criteria.

## Limitations

This study has several limitations. First, the analysis was performed exclusively on video data and did not include synchronized physiological ground-truth signals such as electrocardiography (ECG) or electromyography (EMG), which limits the depth of clinical validation. Although remote photoplethysmographic and oculomotor descriptors correlate with physiological phenomena, their absolute accuracy in distinguishing synthetic from authentic conditions remains indirect and should be verified against concurrent biosignal recordings. Second, the DeepFake RealWorld (DFRW) dataset, while designed to reproduce real-world degradations, does not provide sufficient coverage to establish demographic fairness or representativeness across clinical acquisition environments. In particular, variation related to ethnicity, age, skin appearance, recording context, and telemedical workflow conditions may not be adequately captured. Although the present framework partially mitigates such variability through relative temporal descriptors, shape-based features, and normalization to authentic reference distributions, these design choices do not substitute for dedicated subgroup analysis. Future research should therefore incorporate demographically stratified and clinically acquired multimodal datasets with verified physiological ground-truth, including synchronized ECG, EMG, and respiratory signals, together with explicit subgroup benchmarking. Accordingly, the present study should be understood as incorporating only preliminary mitigation measures, not as resolving the question of demographic representativeness or clinical-environment validity. This limitation is central to the interpretation of the findings, because the observed performance may partly reflect the composition, degradation structure, and acquisition characteristics of the DFRW dataset. Third, the study did not include a direct quantitative head-to-head benchmark against representative prior deepfake detectors under a shared evaluation protocol on the DFRW dataset. Therefore, the present work should be interpreted as introducing a complementary and interpretable biomarker-based framework rather than as establishing numerical competitiveness or superiority over prior methods. Comparisons with previously published methods should thus be regarded as contextual rather than definitive, and future work should include unified benchmarking against both deep learning-based and physiology-oriented detectors. Fourth, although the present protocol includes internal statistical validation and degradation-stratified robustness assessment, it does not include external validation across independent datasets or populations, nor does it yet include a dedicated external held-out benchmark or an independently released raw dataset. As a result, full independent reproduction and external validation remain constrained at the raw-data level. Future work should therefore include formal cross-dataset validation, population-level replication on demographically or clinically stratified cohorts, and, where legally feasible, broader release of reproducible evaluation assets. Accordingly, claims regarding robustness and practical real-world implementation should be interpreted as limited to the evaluated degradation scenarios and as prospective rather than deployment-level conclusions. Fifth, despite discussing possible relevance to telemedicine and imaging quality assurance, the study did not include clinical datasets, clinician interpretation tasks, patient-level outcomes, or prospective validation in diagnostic workflows. Therefore, the clinical relevance of the proposed biomarkers should currently be regarded as conceptual rather than validated for routine medical use. Accordingly, any reference to telemedicine, dentistry, or clinical diagnostics in the present manuscript should be interpreted as prospective application context rather than evidence of clinically validated deployment.

## Conclusion

This study indicates that physiological and morphometric anomalies may serve as interpretable and degradation-aware descriptor-level indicators for differentiating synthetic media within the evaluated DFRW dataset and degradation conditions. The dual-layer framework connects digital security engineering with biomedical signal analysis by integrating measurable biological and geometric principles like photoplethysmographic variability, oculomotor dynamics, and topological consistency. The results obtained on the DeepFake RealWorld (DFRW) dataset suggest that these interpretable descriptors can maintain measurable discrimination under the compression and noise conditions evaluated in the present study, achieving mean Δp ≈ 0.21 and PR up to 4.7, while the added benchmarking metrics, including sensitivity, specificity, accuracy, F1 score, and ROC AUC, facilitate contextual comparison with existing detection studies. Physiological features may provide rapid, low-cost triage signals, while morphometric and topological features may support more detailed forensic review, making the proposed framework a candidate interpretable complementary layer within broader detection pipelines rather than a validated, stand-alone, or deployment-ready detection system. Beyond their forensic relevance, the findings suggest possible future relevance to clinical quality assurance, particularly in telemedicine and dentistry, where authenticity and physiological plausibility of visual data may influence downstream interpretation; however, no clinical validation was performed in the present study. Likewise, no claim of demographic neutrality, cross-dataset generalizability, or representativeness across clinical environments should be inferred from the present dataset. Future work will focus on expanding the dataset to DFRWv2 (>500,000 clips), incorporating diffusion and 3D generative models, and developing standardized protocols for rolling benchmarks and XAI-based uncertainty reporting. In summary, the integration of biologically meaningful and geometrically grounded descriptors offers a methodological pathway for future research on trustworthy multimedia forensics and medically relevant AI safety, while requiring external validation, prospective workflow assessment, and independent replication before any claim of operational effectiveness can be made.

## Data Availability

The raw data supporting the conclusions of this article will be made available by the authors, without undue reservation.
